# Weavable asymmetric carbon nanotube yarn supercapacitor for electronic textiles†

**DOI:** 10.1039/c8ra01384e

**Published:** 2018-04-09

**Authors:** Changsoon Choi, Jong Woo Park, Keon Jung Kim, Duck Weon Lee, Mônica Jung de Andrade, Shi Hyeong Kim, Sanjeev Gambhir, Geoffrey M. Spinks, Ray H. Baughman, Seon Jeong Kim

**Affiliations:** Center for Self-powered Actuation, Department of Biomedical Engineering, Hanyang University Seoul 04763 Korea sjk@hanyang.ac.kr; Division of Smart Textile Convergence Research, Daegu Gyeongbuk Institute of Science and Technology (DGIST) Daegu 42988 Korea; The Alan G. MacDiarmid NanoTech Institute, University of Texas at Dallas Richardson TX 75083 USA; Intelligent Polymer Research Institute, ARC Centre of Excellence for Electromaterials Science, University of Wollongong Wollongong NSW 2522 Australia

## Abstract

Asymmetric supercapacitors are receiving much research interests due to their wide operating potential window and high energy density. In this study, we report the fabrication of asymmetrically configured yarn based supercapacitor by using liquid-state biscrolling technology. High loading amounts of reduced graphene oxide anode guest (90.1 wt%) and MnO_2_ cathode guest (70 wt%) materials were successfully embedded into carbon nanotube yarn host electrodes. The resulting asymmetric yarn supercapacitor coated by gel based organic electrolyte (PVDF-HFP-TEA·BF_4_) exhibited wider potential window (up to 3.5 V) and resulting high energy density (43 μW h cm^−2^). Moreover, the yarn electrodes were mechanically strong enough to be woven into commercial textiles. The textile supercapacitor exhibited stable electrochemical energy storage performances during dynamically applied deformations.

## Abstract 2

Yarn type asymmetric supercapacitor with high weight ratio of rGO in anode for wearable electronics.

## Graphical abstract

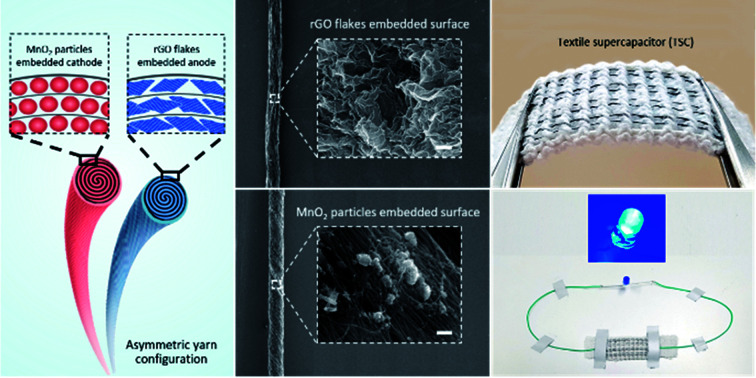

There is an especially important need for weavable yarn-based supercapacitors that can be used in electronic textiles. Yarn-based batteries can provide high specific energy storage capabilities, but their typically low charge and discharge rates are a problem for rapidly storing the energy generated by energy harvesters operating at the frequencies of body motion, or delivering high electrical power when needed.

Yarn or fiber based supercapacitors have advantages over conventional three- or two-dimension (3D, 2D) energy storage devices for powering wearable electronics, which can include micron-scale diameters, light weight, flexibility or stretchability, and weavability into textiles.^1–7^ However, the energy storage densities of supercapacitors are lower than for the best batteries. In order to achieve high energy storage density for yarn or fiber based supercapacitors, previous research has been conducted in two directions: one is to increase the capacitance (*C*) of the device by introducing pseudocapacitive materials, while the other is to widen the voltage window (*V*) of electrochemical operation by using asymmetric electrodes. Since the stored electrical energy is given by *E* = 1/2*CV*^2^, both strategies are important.

Environmentally friendly, cost-effective, highly performing metal oxides (MnO_2_)^8,9^ or various conducting polymers (*e.g.*, poly(3,4-ethylenedioxythiophene) (PEDOT), polyaniline (PANI), polypyrrole (PPy))^2,10–14^ have been extensively studied as pseudocapacitive additives to dramatically improve the charge storage capability of 1D supercapacitors. Asymmetrically configured 1D supercapacitors used active materials like graphene, carbon nanotubes (CNTs), and PPy for anode yarns and materials like MnO_2_, MoS_2_, Ni(OH)_2_, Co_3_O_4_ for cathode yarns, resulting in voltage windows between 1.5 V and 1.8 V.^15–22^

In this study, we realized fiber supercapacitors having both high specific capacitances and increased potential windows. The first utilized strategy was to trap pseudocapacitive guest materials within vascular, high electrical conductivity networks of twist-spun CNT yarns, which maximized the weight percent of the guest without significantly hindering accessibility of the electrolyte to the guest. The second strategy was to use an asymmetric electrode configuration that comprised a MnO_2_ containing cathode yarn and a reduced graphene oxide (rGO) containing anode yarn.

Specifically, both anode and cathode yarns were made using a novel liquid-state biscrolling technology, which embedded 90 wt% rGO flakes as the active material in the anode yarn and 70.5 wt% MnO_2_ nanoparticles as the active material in the cathode yarn. Despite the low weight percent of the CNT host, these guests were firmly trapped in the scrolled CNT galleries of this host. This device design resulted in an aqueous-gel-electrolyte-coated, solid-state, asymmetric supercapacitor that had a 2.1 (V) working potential and a high areal energy density of 30.1 μW h cm^−2^. Moreover, use of an organic gel electrolyte coating on the electrode yarns, extended the working voltage range up to 3.5 V, and increased the areal energy density up to 43 μW h cm^−2^, which is much higher than previously reported for asymmetric yarn supercapacitors. Despite the high loading of brittle pseudocapacitive materials, the electrolyte-coated yarns could be inserted into a knitted structure textile. A textile patch, comprising eight woven supercapacitor yarns that were connected in-series or in-parallel, was used to light a blue LED for 300 seconds.

Fig. 1A schematically illustrates a solid-state, asymmetric yarn supercapacitor comprising anode and cathode CNT yarns containing embedded rGO flake and MnO_2_, respectively. These guest-embedded electrodes were prepared by using a powerful technology called biscrolling.^23^ CNT sheet stacks for the yarn host were drawn from a forest of vertically aligned CNT forest that was fabricated by chemical vapor deposition. Electrochemically active guest materials (rGO flake and MnO_2_ for anode and cathode, respectively) were dispersed in ethanol solution and drop casted onto a stack of CNT sheet ribbons. Twist insertion into guest-covered sheet ribbon stacks provided the biscrolled yarn electrodes which contain embedded guest materials. The loading level of active guest materials was roughly controlled by adjusting particle concentration in the dispersion.

**Fig. 1:**
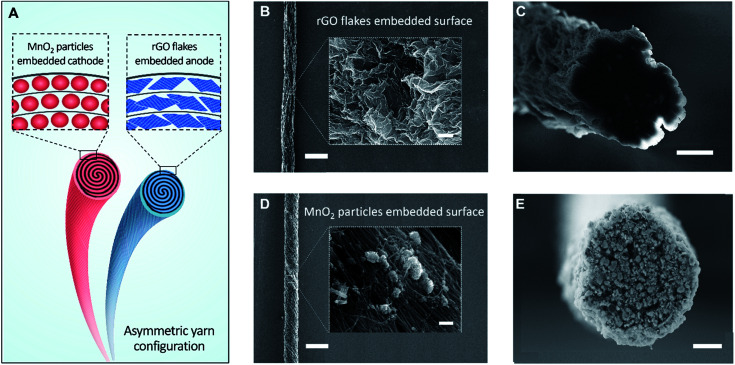
(A) Illustration of a solid-state, asymmetrically configured yarn supercapacitor. Both embedded yarn electrodes were fabricated by liquid-state biscrolling process. SEM images of the surface of a 90 wt% rGO embedded yarn anode at (B) low (scale bar = 300 μm), high magnification (inset, scale bar = 600 nm), and (C) its cross-section (scale bar = 10 μm). SEM images of the surface of a 70 wt% MnO_2_ embedded yarn cathode at (D) low (scale bar = 300 μm), high magnification (inset, scale bar = 600 nm), and (E) its cross-section (scale bar = 15 μm).

Scanning electron microscope (SEM) images of 90 wt% rGO embedded yarn surface and its cross-section are shown in Fig. 1B and C, respectively. The rGO flakes were obtained from chemically reduced graphene oxide sheet, as previously reported.^24^ Although most of the yarn guest is biscrolled into the yarn volume, the structure of guest on the yarn surface provides some indication of the guest structure within the yarn. The indicated flower-like crumbled structure surface of the rGO will enhance the diffusion rate within the yarn and avoid platelet stacking, which would reduce the surface area that is available during fast charge and discharge. SEM images of 70 wt% MnO_2_ embedded yarn surface and cross section are also shown in Fig. 1D and E, respectively.

The electrochemical performance of a symmetric-electrode, electrochemical capacitor is shown in Fig. 2, wherein each yarn is a biscrolled yarn electrode containing 90.1 wt% rGO flakes, which is over coated by and infiltrated with an aqueous polyvinyl alcohol (PVA)–LiCl gel electrolyte. Rectangular cyclic voltammetry (CV) curves (from 10 to 100 mV s^−1^ voltage scan rate) and triangular galvanostatic charge/discharge curves (from 0.5 to 5 mA cm^−2^ current density) were obtained, as shown in Fig. 2A and B, respectively. The CV curves did not show any peaks that can be associated with a faradic redox reaction. Linear and areal capacitances, normalized to the total length and external surface area of a single electrode, are shown as a function of *versus* scan rate in Fig. 2C. The length and surface area of single GO biscrolled yarn electrode were 1 cm and 0.1 cm^2^, respectively. For application in textiles, the area-normalized capacitance is especially important, since it helps define how much energy can be stored in a specific textile area.

**Fig. 2:**
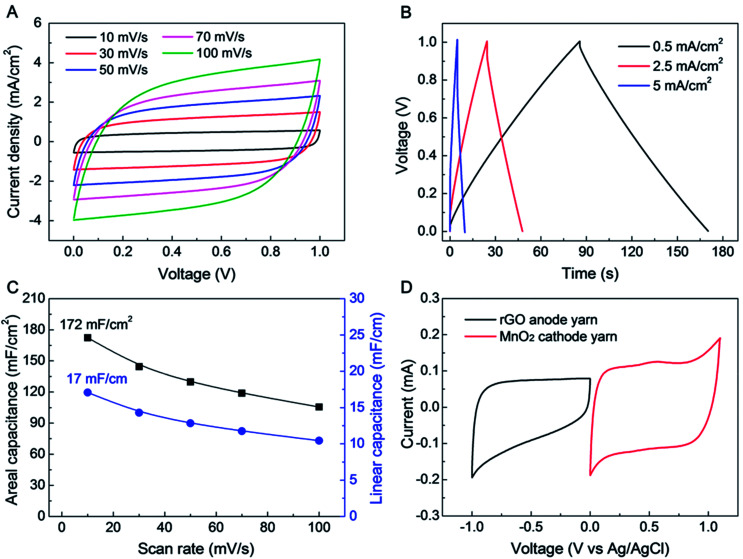
Electrochemical performance of symmetric supercapacitor comprising 90 wt% rGO embedded yarn electrodes and an aqueous PVA–LiCl gel electrolyte. (A) CV curves measured at various scan rates (from 10 to 100 mV s^−1^) and (B) galvanostatic charge/discharge curves (for currents between 0.5 and 5 mA cm^−2^). (C) Calculated areal and linear capacitances (based on CV curves) *versus* scan rate. (D) Combined CV curves for anode and cathode yarns, which cover the voltage range from −1 to 1 V. These CV curves were measured in a three electrode system, using Ag/AgCl as reference electrode and Pt mesh as counter electrode.

Since the areal capacitance of the biscrolled rGO yarn supercapacitor is roughly proportional to the rGO guest loading level (Fig. S2†), the highest charge storage capability was obtained from the supercapacitor yarn having the highest content of rGO (90.1 wt%). This symmetric-electrode supercapacitor provided linear and areal capacitances of 17 mF cm^−1^ and 172 mF cm^−2^, respectively, which were calculated from the CV curve measured at 10 mV s^−1^ scan rate. These presently realized specific capacitances normalized with respect to a single electrode are significantly higher than previously reported for all-carbon-based supercapacitors comprising coaxial CNT fibers (0.029 mF cm^−1^, 8.66 mF cm^−2^),^25^ rGO/CNT composite wires (0.027–0.35 mF cm^−1^, 4.97 mF cm^−2^),^26,27^ ordered mesoporous carbon (OMC) biscrolled fibers (1.91 mF cm^−1^, 39.7 mF cm^−2^),^28^ and even comparable coaxial wet-spun rGO/CNT composite yarns (5.3 mF cm^−1^, 177 mF cm^−2^).^29^ Such a high energy storage capability was achieved as a consequence of the high loading of rGO guest flakes that was enabled by biscrolling, as well as the structure of the crumbled structure of the rGO, which enabling them to maintain high electrochemically accessible surface area at high guest loading.

Further improvement in the performance of a yarn supercapacitor can be obtained by combining the above biscrolled rGO/CNT electrode with a biscrolled MnO_2_/CNT cathode^1^ to make an asymmetric yarn supercapacitor. To explore this opportunity, CV curves for 90 wt% rGO embedded anode and 70 wt% MnO_2_ embedded cathode were measured in 0.1 M Na_2_SO_4_ solution (Fig. 2D), using a three electrode system, with Pt mesh, and Ag/AgCl as counter, and reference electrodes, respectively. The MnO_2_ loading level of 70 wt% was chosen to balance the charge between anode and cathode yarns when the loading level of the rGO electrode was 90 wt%.

The electrochemical performance of an asymmetric supercapacitor comprising a 90.1 wt% rGO embedded yarn anode, a 70 wt% MnO_2_ embedded yarn cathode, and a PVA–LiCl based aqueous gel electrolyte coating is shown in Fig. 3. Fig. 3A shows that supercapacitor-like CV curves were obtained for voltage scans up to 2.1 V, and there is no evidence of a faradic redox peak. Galvanostatic charge/discharge curves also showed a stable triangular shape up to 2.1 V, with a small IR drop of 47 mV at 1.2 mA cm^−2^ current density (Fig. 3B). The CV curves (0 to 2.1 V) for the asymmetric supercapacitor retained a stable rectangular shape when scan rate increased from 10 to 500 mV s^−1^, retaining 61.8% initial capacitance (Fig. 3C, S4C†). The volumetric and areal capacitances for this asymmetric supercapacitor (measured for up to 2.1 V applied potential) were 57.2 F cm^−3^ and 322.4 mF cm^−2^, respectively, for a voltage scan rate of 10 mV s^−1^ (Fig. S4†). Also, this asymmetric supercapacitor exhibited excellent capacitance retention during 1000 charge/discharge cycles at 200 mV s^−1^ scan rate (Fig. S5†).

**Fig. 3:**
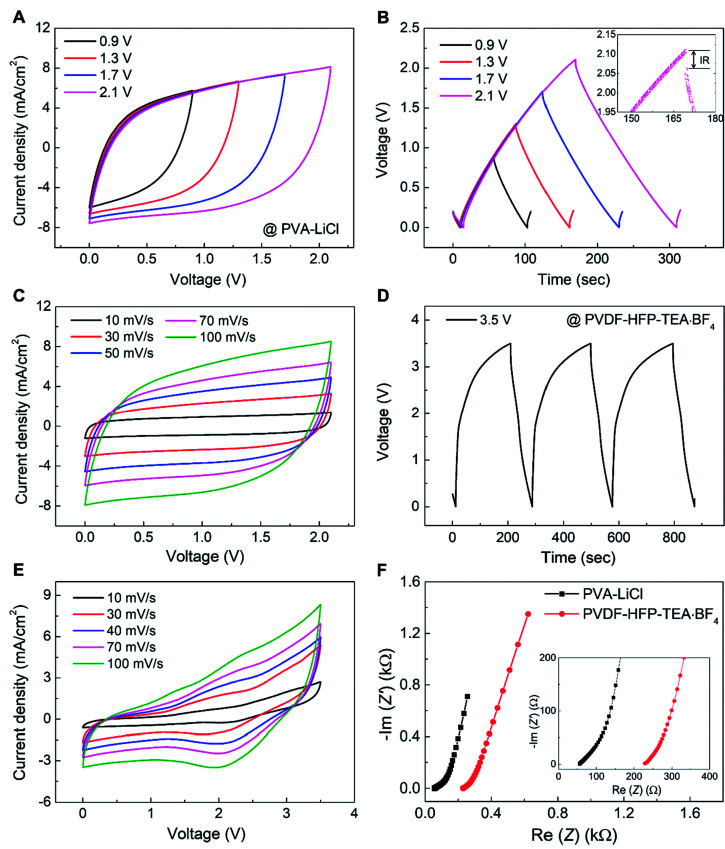
Electrochemical performance of an asymmetric supercapacitor comprising a 90 wt% rGO embedded yarn anode, a MnO_2_ nanoparticle embedded yarn cathode and an aqueous PVA–LiCl gel electrolyte or a polycarbonate-containing PVDF-HFP-TEA·BF_4_ organic gel electrolyte. (A) CV curves at 100 mV s^−1^ scan rate for differing maximum applied voltages (from 0.9 V to 2.1 V) when using a PVA–LiCl gel electrolyte. (B) Galvanostatic-charge/discharge curves (at 1.2 mA cm^−2^ current density) measured over voltage window of from 0.9 to 2.1 V. (C) CV curves measured at various scan rates (10–100 mV s^−1^) when using a PVA–LiCl gel electrolyte. (D) Galvanostatic-charge/discharge curves (at 1.6 mA cm^−2^) when using a PVDF-HFP-TEA·BF_4_ based organic gel electrolyte. (E) CV curves up to 3.5 V for various voltage scan rates when using PVDF-HFP-TEA·BF_4_ based organic gel electrolyte. (F) Comparison of results from electrochemical impedance spectroscopy when using an aqueous PVA–LiCl electrolyte and a PVDF-HFP-TEA·BF_4_ based organic electrolyte.

For further increase of the working voltage window, we introduced a poly(vinylidenefluoride-hexafluoropropylene) (PVDF-HFP) gel-based tetraethylammonium tetrafluoroborate (TEA·BF_4_) organic electrolyte in propylene carbonate (PC) as a higher redox stability electrolyte for the rGO embedded yarn anode and MnO_2_ embedded yarn cathode asymmetric supercapacitor described above.^30,31^ The galvanostatic charge/discharge curves showed a stable triangular shape up to 3.5 V for a current density of 1.6 mA cm^−2^ (Fig. 3D). The organic-gel-electrolyte-coated asymmetric supercapacitor was investigated over a 3.5 V range, for voltage scan rates from 10 to 100 mV s^−1^ (Fig. 3E). The thereby determined volumetric and areal capacitances for 10 mV s^−1^ scan rate were 34 F cm^−3^ and 171 mF cm^−2^, respectively. Moreover, the capacitance measured at 10 mV s^−1^ was retained 60% when scan rate increased to 100 mV s^−1^. Electrochemical impedance spectroscopy for PVA–LiCl and PVDF-HFP-TEA·BF_4_ coated asymmetric supercapacitors indicates low equivalent series resistance, as shown in Fig. 3F.

The scan rate dependence of areal and linear capacitances for asymmetric supercapacitors using PVDF-HFP-TEA·BF_4_ and PVA–LiCl electrolytes are compared in Fig. 4A. Although the organic electrolyte supercapacitor has a wider working voltage range, the specific capacitances were much lower than for the aqueous PVA–LiCl gel based supercapacitor. The reduced charge storage capability for the organic-electrolyte-based supercapacitor possibly results from larger ion size for this electrolyte.

**Fig. 4:**
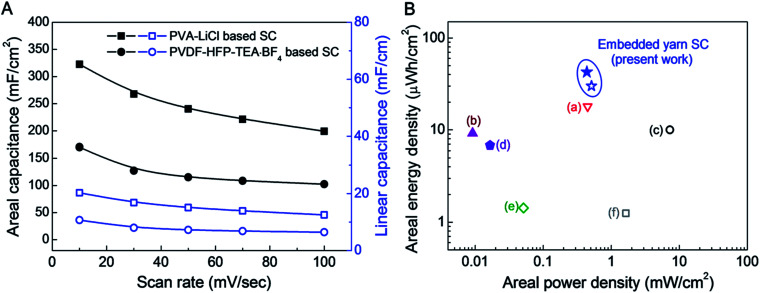
(A) Areal and linear capacitances for an asymmetric supercapacitor comprising a 90 wt% rGO embedded yarn anode and a MnO_2_ nanoparticle embedded yarn cathode when using either an aqueous PVA–LiCl gel electrolyte or a PVDF-HFP-TEA·BF_4_ based organic gel electrolyte. (B) A Ragone plot of areal energy density *versus* areal power density for asymmetric rGO/MnO_2_ embedded yarn supercapacitors, based on the total surface area of the complete supercapacitor, including embedded yarn and PVDF-HFP-TEA·BF_4_ or PVA–LiCl based gel electrolytes. The maximum measured areal energy densities of presently investigated PVDF-HFP-TEA·BF_4_ organic and PVA–LiCl based aqueous gel electrolytes coated asymmetric embedded yarn supercapacitors are 30.1 and 43 μW h cm^−2^, respectively. Previously published data for assymetric yarn or fiber supercapacitors are included for comparison: (a) CNT/MnO_2_ based stretchable asymmetric fibers (18.9 μW h cm^−2^),^18^ (b) rGO/MnO_2_/PPy yarns (9.2 μW h cm^−2^),^28^ (c) NiOH/MnO_2_ asymmetric yarns (10 μW h cm^−2^),^12^ (d) hollow rGO fibers (6.8 μW h cm^−2^),^29^ (e) MnO_2_ nanosheet decorated asymmetric carbon fibers (1.428 μW h cm^−2^),^17^ and (f) MnO_2_ coated stretchable, asymmetric CNT wires (1.25 μW h cm^−2^).^15^

A Ragone plot of areal energy density *versus* areal power density for asymmetric rGO/MnO_2_ embedded yarn supercapacitors (based on the total surface area of the complete supercapacitor, including embedded yarns and PVDF-HFP-TEA·BF_4_ or PVA–LiCl based electrolyte) is shown in Fig. 4B. Previously published data for yarn or fiber supercapacitors are included for comparison. The maximum measured areal energy densities of the presently investigated asymmetric supercapacitors based on PVDF-HFP-TEA·BF_4_ organic gel and PVA–LiCl based aqueous gel electrolytes are 30.1 and 43 μW h cm^−2^, respectively (normalized by total device including both two electrodes and electrolyte coating). These energy densities are higher than for previous 1D supercapacitors, which comprise (a) CNT/MnO_2_ based stretchable asymmetric fibers (18.9 μW h cm^−2^),^22^ (b) rGO/MnO_2_/PPy yarns (9.2 μW h cm^−2^),^11^ (c) NiOH/MnO_2_ asymmetric yarns (10 μW h cm^−2^),^16^ (d) hollow rGO fibers (6.8 μW h cm^−2^),^10^ (e) MnO_2_ nanosheet decorated asymmetric carbon fibers (1.428 μW h cm^−2^),^21^ and (f) MnO_2_ coated stretchable, asymmetric CNT wires (1.25 μW h cm^−2^).^19^Table 1 further compares the linear, areal, and volumetric energy densities of the presently reported asymmetric biscrolled yarn supercapacitors with results in the literature for other 1D asymmetric supercapacitors.

**Table 1:** Comparison of specific energies for present and prior-art fiber or yarn based asymmetric supercapacitors

Anode/cathode electrode (ref. no.)	Voltage [V]	*E*_L_ [μW h cm^−1^]	*E*_A_ [μW h cm^−2^]	*E*_V_ [mW h cm^−3^]
rGO/MnO_2_ embedded CNT yarn supercapacitor (present work)	3.5^a^	4.5	43	5
	2.1^b^	5.5	30.1	3.8
MnO_2_ nanosheet/carbon fiber (21)	1.5	0.216	1.43	—
MnO_2_/CNT fiber (19)	1.5	0.047	1.25	1.57
MnO_2_/CNT core-sheath fiber (22)	1.5	6.2	18.9	2.98
Ni(OH)_2_/OMC^c^ based micro fiber (16)	1.5	—	10	2.16
MnO_2_/N-doped CNT fiber (15)	1.8	—	—	5
Co_3_O_4_ nanowire/graphene fiber (17)	1.5	—	—	0.62

a TEA·BF_4_-PC-(PVDF-*co*-HFP).

b LiCl–PVA gel electrolyte.

c OMC: ordered mesoporous carbon.

The goal of the following investigation of a textile based supercapacitor (TSC) is to show that supercapacitor performance does not degrade when supercapacitor yarns are deployed in a very stretchable fabric, even though the mechanical properties of the supercapacitor yarns and fabric are quite different. The commercial textile used had a mock rib structure, which can be used for a strong, elastic hem (such as edges of gloves). Eight 200 μm-diameter yarn electrodes (four biscrolled rGO anode yarns and four biscrolled MnO_2_ cathode yarns) were inserted into the textile (to replace eight elastic yarns in the mock rib stitch), as shown in Fig. 5A. Despite high loadings of guest powders (90 wt% rGO for the anode yarn and 70 wt% MnO_2_, for the cathode yarn) the yarn electrodes were sufficiently strong to be easily inserted into the textile, or even woven, without any additional treatment. Opposite ends of 4 cm long yarn electrodes were electrically connected to 180 μm-diameter Cu wires using silver paste, so that electrical connections could be made, and the woven anode and cathode yarn pairs of a supercapacitor were then jointly coated with aqueous PVA–LiCl gel electrolyte to complete the TSC.

**Fig. 5:**
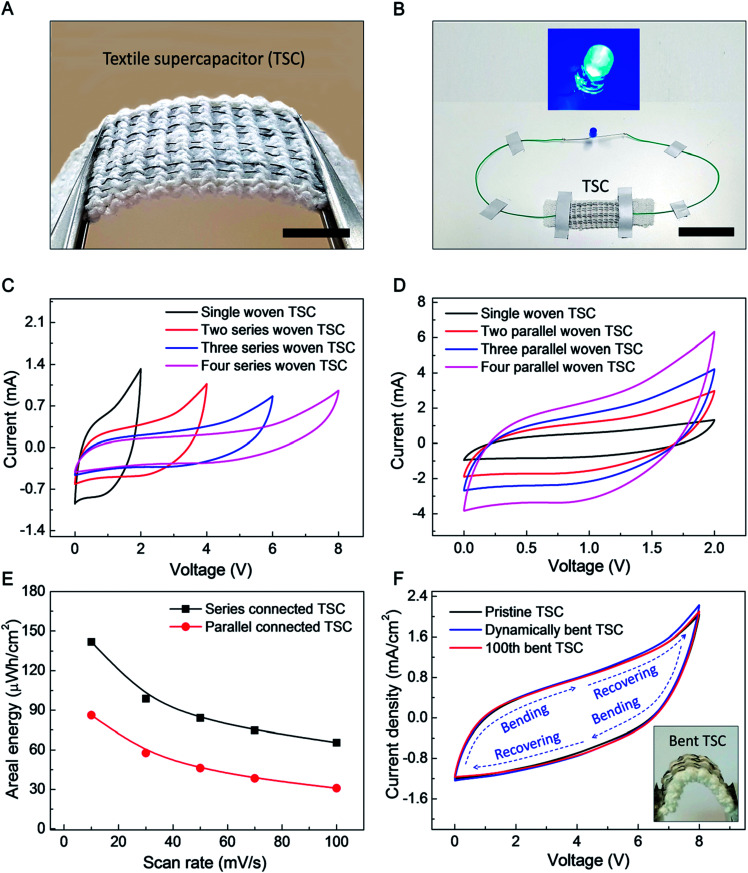
The structure and performance of a textile supercapacitor (TSC) comprising 90 wt% rGO embedded yarn anodes, MnO_2_ nanoparticle embedded yarn cathodes, and an aqueous PVA–LiCl gel electrolyte. (A) Optical image showing four asymmetric yarn supercapacitors sewn into a commercially available knitted textile, each comprising a 4 cm-long rGO embedded anode and a 4 cm-long MnO_2_ embedded cathode (scale bar = 1 cm). (B) Blue LED lightening demonstration by asymmetric TSC (scale bar = 4 cm). CV curves (50 mV s^−1^ scan rate) for (C) series-connected and (D) parallel-connected combinations of from 1 to 4 textile woven supercapacitors. (E) Energy density and specific capacitance of four series and parallel connected TSC *versus* scan rate. Textile surface area occupied by yarn electrodes is used for normalization. (F) The effects on the CV curves of dynamically and cyclically applied bending on a textile containing four series-connected supercapacitors. The CV curves (at 90 mV s^−1^ scan rate) before bending (black line), after 100 bending cycles (red line), and during bending (blue line) are shown.

Four asymmetric woven supercapacitors, each comprising alternating rGO embedded anode and MnO_2_ embedded cathode, were series or parallel direction connected to demonstrate scalability (a 2.5 mm gap between woven supercapacitors was used to prevent ionic currents between supercapacitors which would prohibit their use as series-connected devices). The series connected TSC demonstrated a sufficiently high voltage to power a blue 0.65 W LED for 300 seconds (Fig. S7†), as shown in Fig. 5B.

CV curves for increasing numbers of in-series and in-parallel woven supercapacitors are shown in Fig. 5C and D, respectively. Series connection enabled voltages of up to ∼8 V, while parallel connection enabled discharge current of up to ∼3.2 mA (at 1 V). Based on these CV curves at 10 mV s^−1^ scan rate, the areal energy density and areal capacitance (normalized to total area occupied by the supercapacitors on the textile) for four series-connected TSC are 142 μW h cm^−2^ and 16 mF cm^−2^ and those for four parallel-connected TSC are 86.2 μW h cm^−2^ and 155 mF cm^−2^, respectively. Although the areal capacitance for series-connected TSC is much lower than for parallel connection (as is expected), much higher specific energy was obtained by enlarged operation voltage up to 8 V of series connection. The effects of dynamically and cyclically applied bending on textile containing four series-connected TSC around a mandrel (which results is a bending radius of 4 mm for the supercapacitors in the textile) are described in Fig. 5F. As shown here, 100 bending cycles or bending and releasing the textile during electrochemical cycling produced little change in CV scans.

## Conclusions

In summary, asymmetric yarn supercapacitors which have high energy density and can be woven into textiles were investigated. The rGO embedded CNT yarn electrode which contains 90.1 wt% rGO shows excellent charge storage capability (172 mF cm^−2^) which contributes to enhance overall electrochemical performance of the asymmetric supercapacitors. By combining with MnO_2_ embedded CNT yarn cathode, the wide voltage windows up to 2.1 V at aqueous electrolyte, and 3.5 V at organic electrolyte were obtained. The high areal energy densities measured from all-solid state asymmetric yarn supercapacitors were 30.1 μW h cm^−2^ and 43 μW h cm^−2^ for PVA–LiCl and PVDF-HFP-TEA·BF_4_ gel electrolytes, respectively. Finally, the asymmetric supercapacitors could be woven into a knitted textile to make a TSC, and it shows a promising possibility to be used in electronic textiles by lighting a blue LED.

## Methods

### Preparation of guest-embedded, biscrolled yarn electrochemical electrodes

Narrow sheets of highly oriented carbon multiwalled nanotubes (MWNTs) were drawn from a MWNT forest (∼400 μm high and consisting of ∼12 nm-diameter nanotubes containing ∼9 walls), which was fabricated by a chemical vapor deposition. Nanoflakes of rGO dispersed in dimethylformamide (8 mg ml^−1^) were ultrasonicated (for 1 hour at 150 W using a VCX750 ultrasonic processor from Sonics). After ultrasonication, the rGO dispersion was drop cast on a 5-layer-stack of these forest-drawn MWNT sheets, which were ∼3 cm wide and 20 cm long, and semi-dried for ∼20 minutes. The drop casting and the semi-drying processes were repeated five times. Afterward, the rGO/CNT sheet stack was converted to a biscrolled yarn by inserting ∼3000 turns per meter (per sheet length) using an electrical motor. Commercially available MnO_2_ nanoparticles (rod shape with ∼30 nm diameter and ∼100 nm length from Sigma-Aldrich) were dispersed in ethanol (5 mg ml^−1^) and ultrasonicated, as done for the rGO. After drop casting the as-prepared MnO_2_ dispersion (100 μl cm^−2^) on a four layer CNT sheet stack, the MnO_2_/CNT sheets were twisted to ∼5000 turns per meter using an electrical motor to make the MnO_2_ embedded biscrolled yarn used as a supercapacitor cathode. One end of each electrode was connected to a 180 μm-diameter Cu wire using silver paste for electrochemical performance measurements.

### Supercapacitor assembly

The aqueous PVA–LiCl gel electrolyte was prepared by heating a mixture of 3 g PVA (*M*_w_ 146 000–186 000) and 6 g LiCl in 30 ml deionized water at 90 °C for several hours. The PVDF-HFP-TEA·BF_4_ gel electrolyte was prepared by drying a solution mixture of PVDF-HFP in acetone and TEA·BF_4_ in propylene carbonate (PC) on a slide glass for ∼3 hours. The ratio of PVDF-HFP in acetone and TEA·BF_4_ in PC was 4 : 1. The rGO embedded anode and the MnO_2_ embedded cathode were placed parallel and ∼100 μm apart and then coated with PVA–LiCl (or PVDF-HFP-TEA·BF_4_) gel electrolyte to complete asymmetric yarn supercapacitor fabrication. The TSC was made by removing textile threads from the mock rib structure and then using a needle to sew in their place rGO/CNT embedded yarns and MnO_2_/CNT embedded yarns, which were then pairwise coated with the PVA/LiCl gel electrolyte. The chemicals for electrolyte synthesis were purchased from Sigma-Aldrich and Alfa-Aesar.

### Characterization

Cyclic voltammetry and chronopotentiometry measurements for all investigated supercapacitors were made using a electrochemical analyzer (CHI 627b, CH Instrument). Nyquist curves were measured using another electrochemical analyzer (Reference 600, Gamry Instrument). Scanning electron microscope images of biscrolled yarns were obtained using a Zeiss Supra 40 SEM. The length and weight of each electrode were measured using a digital Vernier caliper (500 series, Mitutoyo) and microbalance (XP6, Meter Toledo), respectively. Yarn diameters were obtained from optical images collected using a microscope with attached camera. X-Ray photoelectron spectroscopy data are collected from an X-ray photoelectron spectrometer (Theta Probe, Thermo Scientific Co.).

### Calculation of electrochemical performance

The capacitances of supercapacitors were calculated from cyclic voltammograms using *C* = *I*/(d*V*/d*t*), where *I* is the discharge current and d*V*/d*t* is the voltage scan rate. The specific areal capacitance for each electrode in a supercapacitor having equal anode and cathode capacitances was calculated using *C*_*A*_ = 4*C*/*A*, where *A* is the total surface area of the anode and cathode. The total length and total volume of electrodes were used to obtain linear capacitance and volumetric capacitance, respectively. The specific energy density was calculated from the equation *E* = 1/2*CV*^2^. Resulting energy densities indicated in Fig. 4B and Table 1 are for a complete supercapacitor (normalized by the total length, surface area, and volume of the embedded electrodes coated with electrolyte).

## Conflicts of interest

There are no conflicts to declare.

## Supplementary Material

RA-008-C8RA01384E-s001
