# Supplementary material for: Weavable asymmetric carbon nanotube yarn supercapacitor for electronic textiles
Source: RSC Adv. 2018 Apr 9;8(24):13112–20. doi: 10.1039/c8ra01384e (PMC9079689; doi:10.1039/c8ra01384e)
Supplement: RA-008-C8RA01384E-s001 [file RA-008-C8RA01384E-s001.pdf]

Electronic Supplementary Material (ESI) for RSC Advances.
This journal is © The Royal Society of Chemistry 2018

(Electronic Supplementary Information)

Weavable Asymmetric Carbon Nanotube Yarn Supercapacitor for Electronic Textiles

Changsoon Choi‡[a,b], Jong Woo Park‡[a], Keon Jung Kim[a], Duck Weon Lee[a], Mônica Jung de Andrade[c], Shi Hyeong Kim[c], Sanjeev Gambhir[d], Geoffrey M. Spinks[d], Ray H. Baughman[c] and Seon Jeong Kim*[a]

[a]Center for Self-powered Actuation, Department of Biomedical Engineering, Hanyang University, Seoul 04763, Korea

[b]Division of Smart Textile Convergence Research, Daegu Gyeongbuk Institute of Science and Technology (DGIST), Daegu 42988, Korea

[c]The Alan G. MacDiarmid NanoTech Institute, University of Texas at Dallas, Richardson, TX 75083, USA

[d]Intelligent Polymer Research Institute, ARC Centre of Excellence for Electromaterials Science, University of Wollongong, Wollongong, NSW, 2522, Australia

* Corresponding author.
E-mail address: sjk@hanyang.ac.kr (S. J. Kim)

‡These authors contributed equally.

Key words: embedded yarn, asymmetric supercapacitor, electronic textile, carbon nanotube, reduced graphene oxide

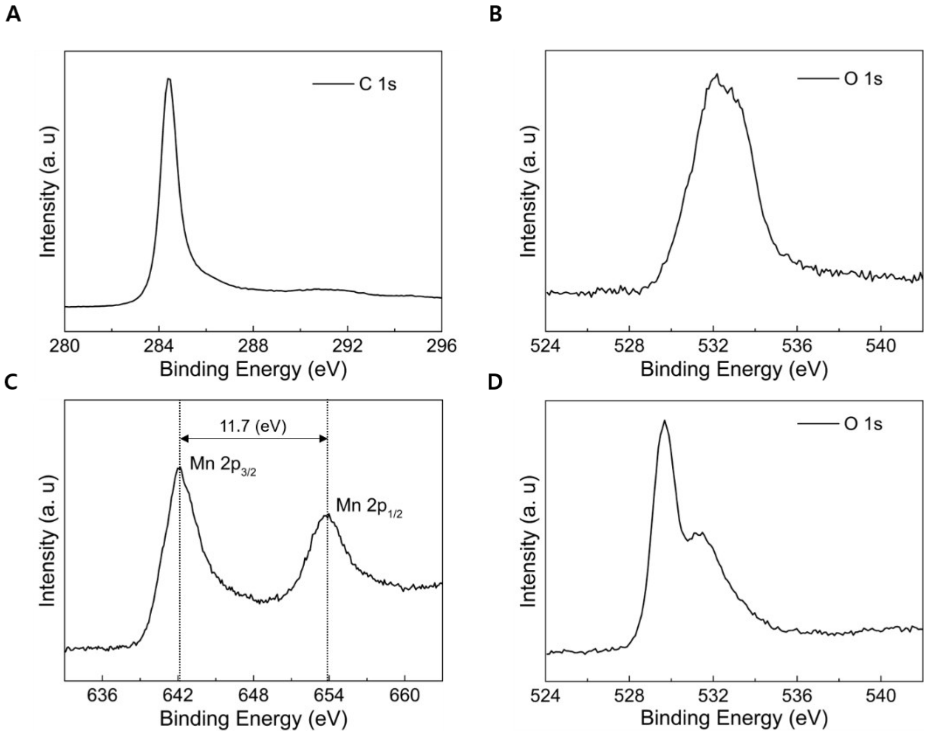

Figure S1. Binding energy data of the 90 wt% rGO embedded CNT yarn surface (A, B) and the 70 wt% MnO_2 embedded CNT yarn surface (C, D) from x-ray photoelectron spectroscopy(XPS). In C 1s component in rGO embedded yarn, the ratio of C-C peak (284.4 eV) is majority and the effect of C-O peak is barely seen due to the vast existence of CNT bundles. However, in O 1s component, it is clearly indicated that of C-OH peak (532.2 eV). In Mn 2p component of the MnO_2 embedded yarn, the binding energy separation between the Mn $2p_{3/2}$ (642 eV) and Mn $2p_{1/2}$ (653.7 eV) peaks is 11.7 eV. Also, O 1s component shows the combination of Mn-O-Mn, Mn-O-H, H-O-H peaks.

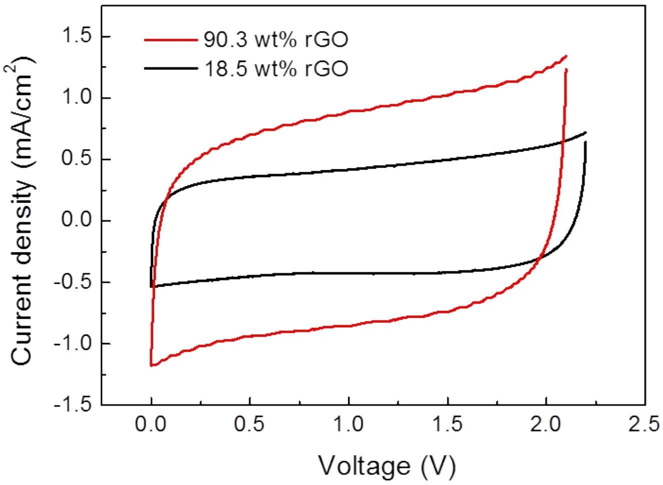

Figure S2. The effect on cyclic voltammetry (CV) curves of increasing the amount of rGO flakes in the biscrolled anode of an asymmetric supercapacitor from 18.5 and 90.3 wt%. The counter electrode was a biscrolled yarn containing 70 wt% MnO_2 and the electrolyte was aqueous polyvinyl alcohol (PVA)-LiCl gel.

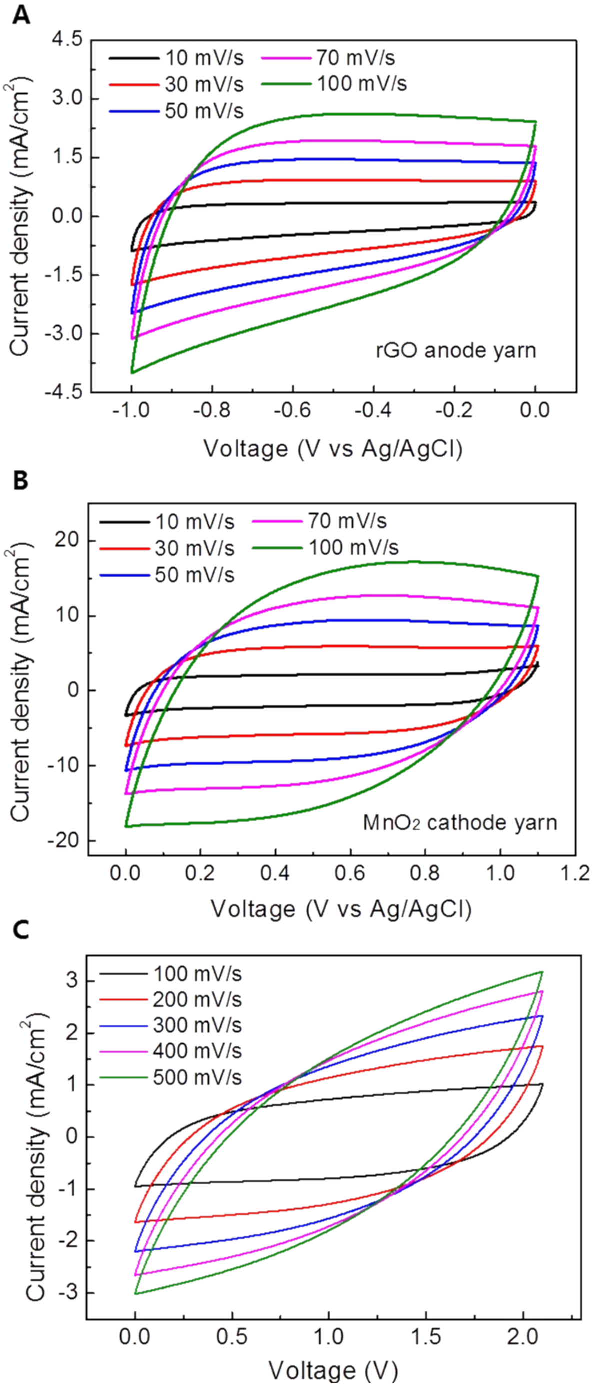

Figure S3. CV curves for different potential scan rates for (**A**) a 90 wt% rGO embedded CNT anode, (**B**) a 70 wt% MnO_2 embedded CNT cathode, and (**C**) an asymmetric supercapacitor comprising these electrodes. The CV curves of (A) and (B) were obtained for a three electrode system (with Pt mesh counter electrode and Ag/AgCl reference electrode), while the CV curves of (C) were obtained for a two electrode system comprising a 90 wt% rGO embedded anode and a 70 wt% MnO_2 embedded cathode, which were coated with and infiltrated with PVA-LiCl gel electrolyte. The electrolyte used for (A) and (B) was 0.1 M Na_2SO_4 liquid electrolyte.

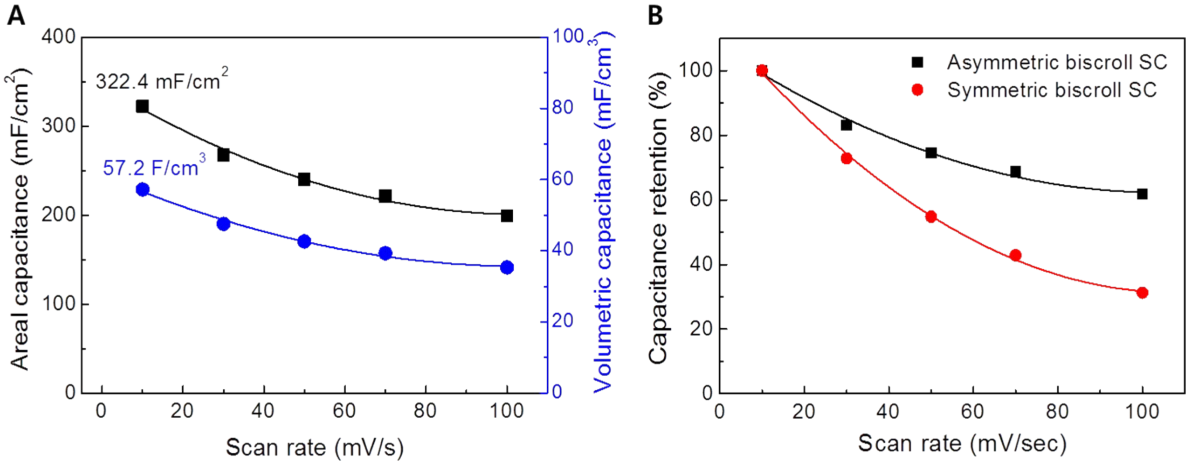

Figure S4. (**A**) Areal and volumetric capacitance versus scan rate for an asymmetric yarn supercapacitor. (**B**) Capacitance retention versus potential scan rate for symmetric and asymmetric yarn supercapacitors. The asymmetric supercapacitor comprised a 90.1 wt% rGO embedded yarn anode, a 70 wt% MnO_2 embedded yarn cathode, and a PVA-LiCl based aqueous gel electrolyte. The symmetric supercapacitor used a 90.1 wt% rGO embedded yarn for both electrodes, as well as the PVA-LiCl based aqueous gel electrolyte. Both the asymmetric and symmetric supercapacitors were two parallel biscrolled CNT yarns.

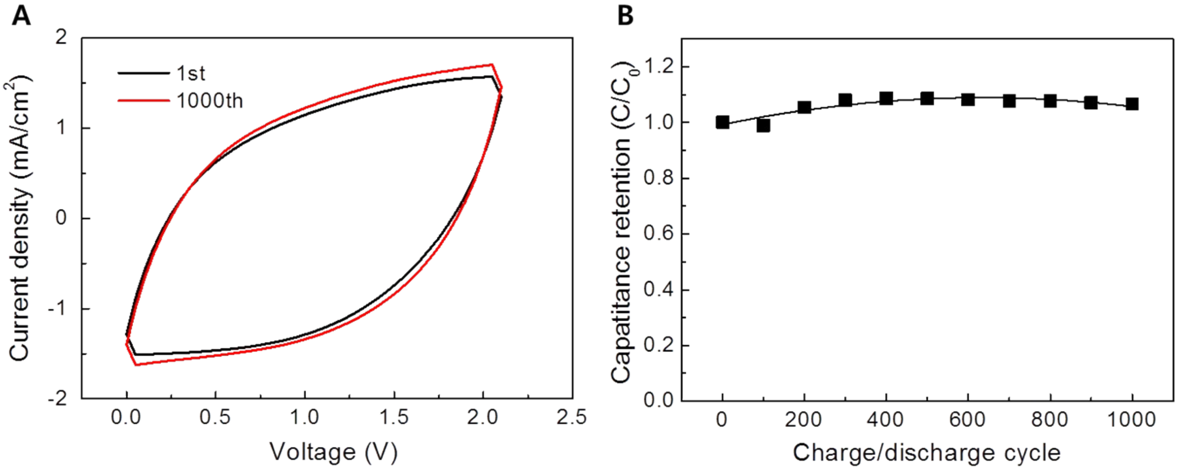

Figure S5. (**A**) CV curves (at 200 mV/s) before and after 1000 charge/discharge cycles for the asymmetric supercapacitor of fig. S3, which comprised a 90.1 wt% rGO embedded yarn anode, a 70 wt% MnO_2 embedded yarn cathode, and a PVA-LiCl based aqueous gel electrolyte. (**B**) capacitance retention versus number of charge/discharge cycles for the asymmetric supercapacitor of (A).

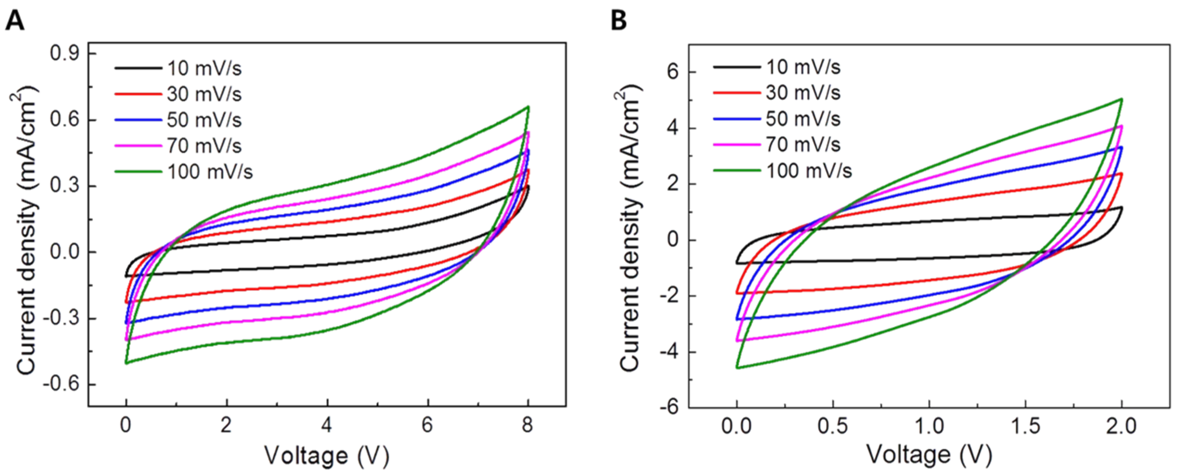

Figure S6. CV curves measured at different scan rates (10-100 mV/s) for (**A**) an asymmetric supercapacitor that uses a propylene carbonate PVDF-HFP- TEA·BF_4 organic gel electrolyte and (**B**) an otherwise nearly identical asymmetric supercapacitor that uses a PVA-LiCl aqueous gel electrolyte. The asymmetric supercapacitors contained a 90.1 wt% rGO embedded yarn anode and a 70 wt% MnO_2 embedded yarn cathode, which were electrolyte coated and plied together. PVDF-HFP is poly(vinylidenefluoride-hexafluoropropylene) and TEA·BF_4 is tetraethylammonium tetrafluouroborate.

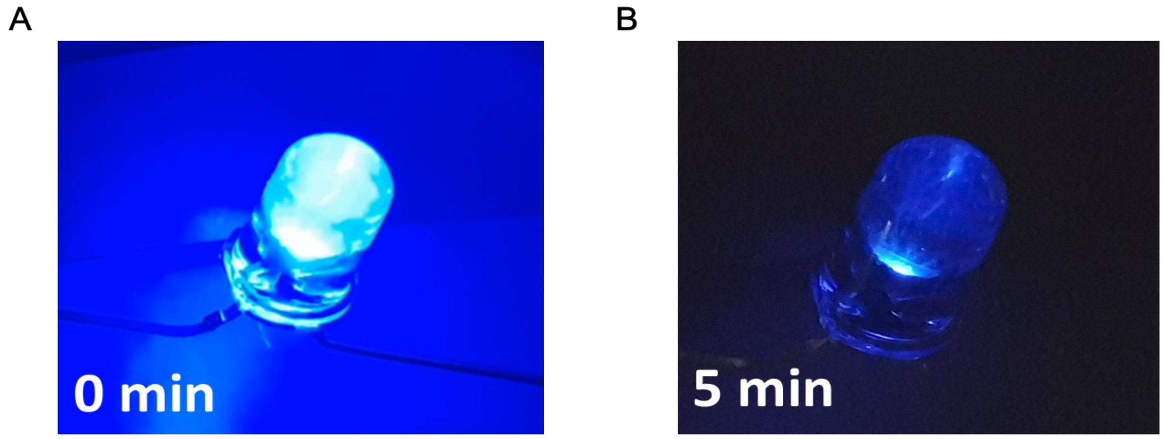

Figure S7. (A) Photograph showing a blue LED that is powered by a textile containing a woven supercapacitor (**B**) Photograph of the blue LED 5 minutes later, when the energy on the textile supercapacitor is nearly exhausted.
